# Protection of Hepatocytes from Cytotoxic T Cell Mediated Killing by Interferon-Alpha

**DOI:** 10.1371/journal.pone.0000791

**Published:** 2007-08-29

**Authors:** Christian B. Willberg, Scott M. Ward, Reginald F. Clayton, Nikolai V. Naoumov, Christopher McCormick, Sandra Proto, Mark Harris, Arvind H. Patel, Paul Klenerman

**Affiliations:** 1 Nuffield Department of Medicine, Oxford University, Oxford, United Kingdom; 2 Medical Research Council Virology Unit, Glasgow, United Kingdom; 3 The University College London Institute of Hepatology, University College London, London, United Kingdom; 4 Faculty of Biological Sciences, Institute of Molecular and Cellular Biology, University of Leeds, Leeds, United Kingdom; 5 Division of Experimental Medicine, University of California at San Francisco, San Francisco, California, United States of America; 6 Princess Alexandra Hospital, Woolloongabba, Australia; 7 Molecular Microbiology and Infection, University of Southampton, Southampton General Hospital, Southampton, United Kingdom; New York University School of Medicine, United States of America

## Abstract

**Background:**

Cellular immunity plays a key role in determining the outcome of hepatitis C virus (HCV) infection, although the majority of infections become persistent. The mechanisms behind persistence are still not clear; however, the primary site of infection, the liver, may be critical. We investigated the ability of CD8+ T-cells (CTL) to recognise and kill hepatocytes under cytokine stimulation.

**Methods/Principle Findings:**

Resting hepatocytes cell lines expressed low levels of MHC Class I, but remained susceptible to CTL cytotoxicity. IFN-α treatment, *in vitro,* markedly increased hepatocyte MHC Class I expression, however, reduced sensitivity to CTL cytotoxicity. IFN-α stimulated hepatocyte lines were still able to present antigen and induce IFN-γ expression in interacting CTL. Resistance to killing was not due to the inhibition of the FASL/FAS- pathway, as stimulated hepatocytes were still susceptible to FAS-mediated apoptosis. *In vitro* stimulation with IFN-α, or the introduction of a subgenomic HCV replicon into the HepG2 line, upregulated the expression of the granzyme-B inhibitor–proteinase inhibitor 9 (PI-9). PI-9 expression was also observed in liver tissue biopsies from patients with chronic HCV infection.

**Conclusion/Significance:**

IFN-α induces resistance in hepatocytes to perforin/granzyme mediate CTL killing pathways. One possible mechanism could be through the expression of the PI-9. Hindrance of CTL cytotoxicity could contribute to the chronicity of hepatic viral infections.

## Introduction

The hepatitis C virus (HCV) is estimated to infect over 100 million people world wide, causing persistent infection in the majority [1]. The cellular immune response is thought to play a major role in the clearance and control of the virus and failure of cellular responses to HCV is a major factor for chronic viral persistence [2]–[4]. The reasons behind this failure are still unclear. One possibility could be the site of infection, the liver itself.

The importance of the liver as a unique site of antigen presentation was initially highlighted by Calne in 1969 [5], [6]. Many of the liver resident cells have been implicated in the regulation of immune responses, including both liver sinusoidal epithelial cells [7] and hepatocytes [8]. However, the precise mechanisms remain to be fully clarified.

Under constant exposure to foreign antigen from the gut, the liver needs to resist or suppress misdirected immune responses. Hepatocytes represent the primary site for replication of several hepatotropic viruses, including HCV. Human hepatocyte expression of MHC Class I has been well documented as low to absent *in vivo* [9]–[13]. Therefore, the ability of hepatocytes to present antigen and act as CTL targets may differ from that of other cells. Understanding T cell-hepatocyte interactions is therefore crucial in defining the mechanisms behind the clearance or persistence of hepatic viral infection.

CTL clearance of viral infection can occur by either the destruction of the infected cells or the up-regulation of antiviral mechanisms within the cells by the expression of IFN-γ. The destruction of infected cells by CTLs can be mediated by either receptor-mediated induction of apoptosis or via the perforin/granzyme pathway. IFN-γ mediated pathways are thought to dominate over cytotoxicity in the clearance of hepatotropic viruses [14], [15], although the mechanisms behind this are not yet clear.

In this study we the modelled CD8+ T cell–hepatocytes using both a traditional hepatocellular carcinoma derived hepatocyte cell line, HepG2, as well as *in vitro* immortalised primary human hepatocyte lines (HHL -6, -16 and -17), previously described [16]. Furthermore, as both HCV and HBV specific CD8+ T Cell responses during chronic infections are weak and potentially dysfunctional [3], [17]–[19], we generated short term CD8+ T cell lines specific for non-hepatotrophic viruses CMV and EBV whose responses are well documented, robust and reproducible. Therefore, CD8+ T cell/hepatocyte interactions were assessed using model hepatocyte cell lines pulsed with the specific cognate peptides and assayed using well-defined T cell populations.

## Results

### Hepatocytes as CTL Targets

CTL recognise potential targets through TCR-MHC Class I interactions. Human hepatocyte expression of MHC Class I has been well documented as low to absent *in vivo* [9]–[13]. Therefore, hepatocytes may represent poor CTL targets and hinder the ability of CTL to clear hepatotrophic viruses, such as HCV. This may contribute to the chronicity of such infections.

MCH Class I expression can be modulated by cytokine stimulation. IFN-α is known to induce the up-regulation of MHC Class I on many cell types including hepatocytes [20]. Moreover, IFN-α represents a major component of the current HCV treatment, as well as being expressed by cells upon viral infection as part of the innate cellular response.

**Figure 1:**
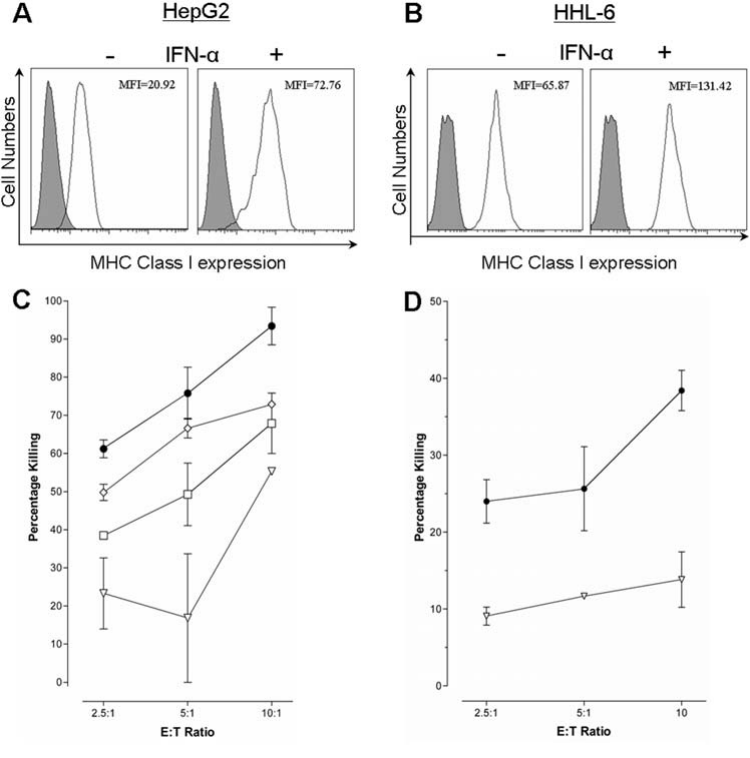
IFN-α reduces hepatocyte sensitivity to CTL cytotoxicity. Hepatocytes have been described as expressing low to no MHC Class I. As expected, IFN-α treatment increased the levels of MHC Class I on HepG2 (A) and HHL (B); filled curve represents an isotype control, the solid line represents the MCH Class I expression. C) HepG2 cells were stimulated for 16 hours with a serial dilution of IFN-α at 0 IU/ml (closed circles), 10 IU/ml (open diamonds), 100 IU/ml (open squares), and 1000 IU/ml (open inverted triangles), prior to cytotoxicity assay with the CTL line 2. Treatment with IFN-α reduced the HepG2 cells sensitivity to CTL cytotoxicity in a dose dependent manner. D) This phenomenon was also found with the novel human hepatocyte cell lines (HHL). HHL-17 cells were either left untreated (closed circles) or stimulated for 16 hours with 1000 IU/ml (open inverted triangles) prior to co-incubation with the CTL line 1.

**Figure 2:**
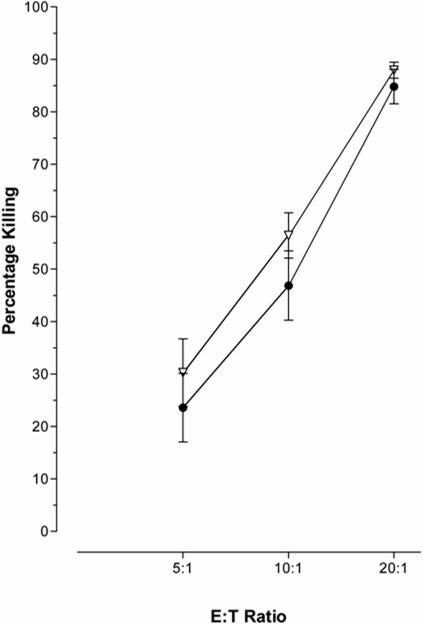
IFN- treatment did not protect a B-cell line. Treatment of a BCL with 1000 IU/ml IFN-α (open inverted triangles) prior to the cytotoxicity assay, did not reduce CTLs ability to kill the treated BCL compared to the untreated BCL (closed circles).

Stimulation of the hepatocellular carcinoma cell line, HepG2, and the novel immortalised human hepatocyte lines (HHL) -6, -16 and -17 (data not shown) with IFN-α resulted in increased MHC Class I expression, figure 1 A–B. However, this was not accompanied by an increase in susceptibility to CTL killing, figure 1 C–D, assessed using the flow cytometry based cytotoxicity VITAL assay [21]. IFN-α treatment rendered the hepatocytes less susceptible to CTL cytotoxicity in a dose dependent manner (Figure 1C). As this phenomenon was also seen in the immortalised HHLs this was not a mechanism of carcinoma escape (Figure 1 D); although it might be a pathway utilised by hepatocellular carcinomas. However, the same effect could not be induced in a B cell line (Figure 2), nor was this observed in keratinocytes (Dr Ogg and Dr Black, Oxford, personal communications).

### Antigen Presentation

**Figure 3:**
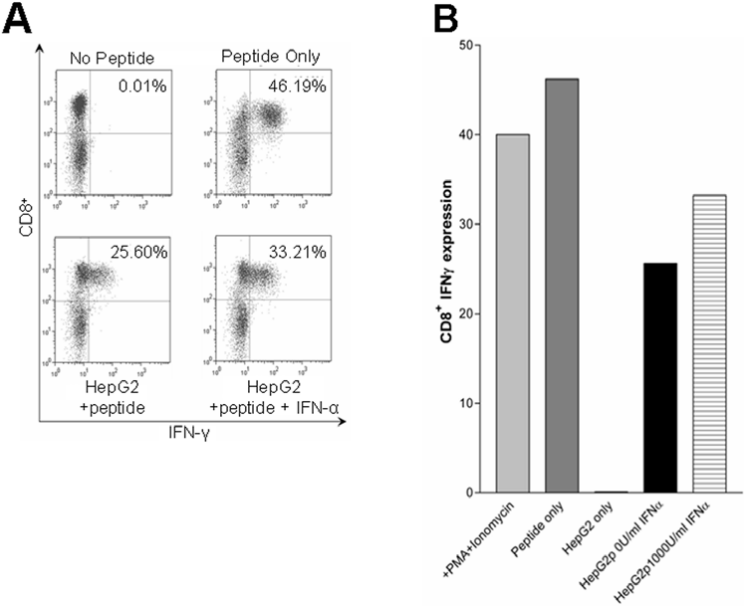
IFN-α stimulated hepatocytes are still able to act as APCs to CTL. HepG2 cells were stimulated for 16 hours with 1000 U/ml IFN-α, or left untreated. The cells were then pulsed with the specific cognate peptide and washed, prior to co-incubation with the CTL line 2. As controls non-peptide-pulsed cells were used, or all PBMC were peptide pulsed or stimulated with PMA/ionomycin. A) The raw FACS data showing the CD8+ expression on the y axis's and IFN-γ on the x axis's. B) The percentage secretion data from such experiment. HepG2p represents peptide pulsed cells with or without IFN-α stimulation as indicated. This data is representative of 4 such experiments.

In order to deduce the mechanism behind the reduced susceptibility of hepatocyte lines to CTL killing, the ability of the hepatocytes to present antigen was first examined. CTL interactions with target cells results in both cytotoxicity and the production of IFN-γ. In order to confirm that IFN-α stimulation did not alter the ability of the hepatocyte lines to present antigen, the levels of IFN-γ induced within interacting CTL was analysed. IFN-γ expression in CTL incubated with IFN-α stimulated and un-stimulated hepatocyte cell lines revealed no detectable difference in the ability of the HepG2 cell line to present antigen (Figure 3). This could also be reproduced with the HHLs, (data not shown).

These data implied that the mechanism behind the reduced killing lay in the ability of the hepatocyte lines to resist CTL cytotoxicity.

### Resistance was Independent of FASL-Induced Apoptosis

**Figure 4:**
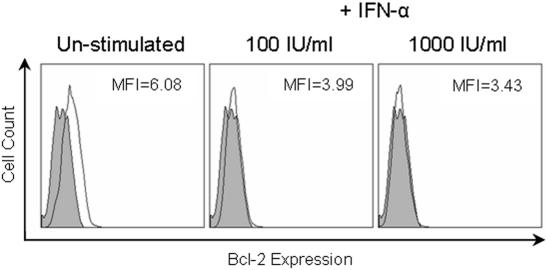
IFN- treatment does not induce Bcl-2 expression. Hepatocyte expression of the anti-apoptotic protein Bcl-2 was assessed by FACS. HHL-17 cells were stimulated with IFN-α at either 0 IU/ml, 100 IU/ml or 1000 IU/ml for 16 hours. Un-stimulated hepatocytes expressed low levels of Bcl-2 (MFI of 6.08), while those treated with either 100 IU/ml or 1000 IU/ml of IFN-α lost expression (MFI of 3.99 and 3.42 respectively); filled curves represent an isotype control, and solid lines represent the Bcl_2_ staining.

Several studies have described hepatocyte sensitivity to FASL-induced apoptosis, both *in vitro* and *in vivo* [22]–[26]. The Bcl protein family represent potent inhibitors of the FASL-mediated apoptotic pathway [27]. The expression of Bcl-2 as well as FAS on the hepatocyte cell lines +/− IFN-α stimulation was analysed (Figure 4). IFN-α stimulation showed no effect on FAS expression on any of the hepatocyte cell lines tested. The expression of Bcl-2 was not seen in all the hepatocyte cell lines. Where Bcl-2 was undetectable IFN-α stimulation had no effect. However, the HepG2 cell line, where Bcl-2 levels were low, IFN-α stimulation led to the reduction of Bcl-2. The decrease in Bcl-2 expression in the IFN-α stimulated cells suggested this molecule is not responsible for resistance to CTL-killing seen.

**Figure 5:**
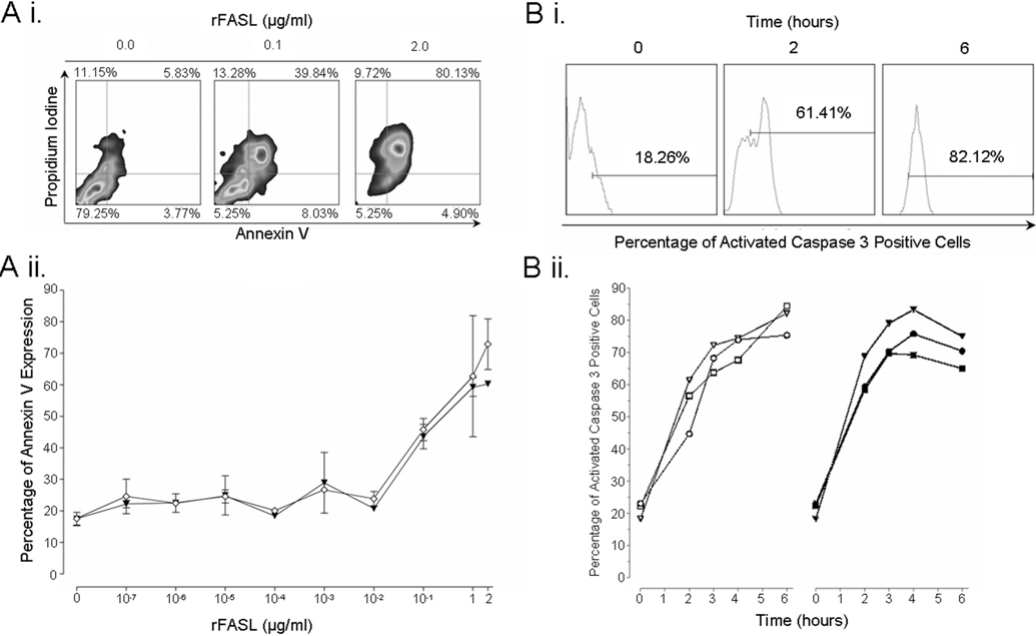
IFN-α treated hepatocytes remain susceptible to FASL-induced apoptosis. Aii) Induction of apoptosis by FASL was assessed across a concentration range. HepG2 cells, either stimulated with IFN-α (1000 IU/ml) (closed inverted triangles) or left un-stimulated (open diamonds), were incubated with cross-linked rFASL at concentrations ranging from 0.1 pg/ml to 2 µg/ml. Apoptosis was assessed by Annexin V binding and propidium iodine (P.I) incorporation. Ai) Raw FACS data is shown. Bii) HepG2 cells, either un-stimulated (open and closed circles), stimulated with 100 IU/ml (open and closed squares) IFN-α or 1000 IU/ml (open and closed inverted triangles) IFN-α, were incubated with 1 µg/ml (left, open symbols) or 2 µg/ml (right, closed symbols) cross-linked rFASL over a time course of up to 6 hours. Apoptosis was assessed by the cytosolic presence of the activated form of caspase 3. Bi) Raw FACS data.

Recombinant FASL (rFASL) with a FLAG motif was used to examine the complete apoptotic pathway in isolation. rFASL was cross-linked on the cell surface by the use of an anti-FLAG antibody, thereby inducing apoptosis. As previously, hepatocyte cell lines were stimulated for 16 hours with IFN-α at varying concentrations prior to incubation with rFASL. IFN-α stimulation of the HepG2 cell line had no protective affect against FASL-induced apoptosis. This was confirmed by Annexin V binding combined with propidium iodine (P.I) incorporation, and by the cytosolic presence of the activated form of caspase 3 (Figure 5A). The duration of the rFAS-FASL interactions also revealed no difference between IFN-α stimulated and un-stimulated cells (Figure 5B).

### FAS^−/−^ Human Hepatocyte Line

**Figure 6:**
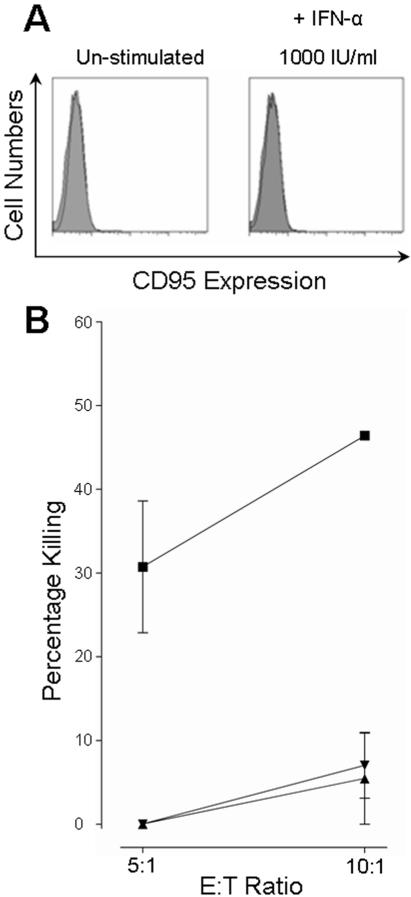
A FAS negative hepatocyte line showed almost complete resistance to CTL killing. A) The lack of FAS expression on the HHL-16 cells was confirmed by FACS analysis in both un-stimulated and IFN-α stimulated cells. FAS expression was negative under both conditions, mean fluorescence intensity of 4.60 and 4.34 respectively (open curves), and never higher than an isotype control (filled curve). B) Under normal conditions (closed squares) the HHL-16 cells were able to act as targets for CTL cytotoxicity after 4 hours co-incubation. However, stimulation with IFN-α, either at 100 IU/ml (closed triangles) or at 1000 IU/ml (closed inverted triangle), reduced killing below 10% even at the higher E:T ratio.

The lack of resistance to FASL-induced apoptosis in IFN-α stimulated hepatocytes was further confirmed in the human hepatocyte line, HHL-16, which was shown to be FAS negative (Figure 6A). Consistent with this, rFASL did not induce apoptosis in this cell line (data not shown). Un-stimulated HHL-16 cells were readily killed by CTL (Figure 6B). However, after stimulation with IFN-α, at either 100 IU/ml or 1000 IU/ml, killing could only be observed at the higher E:T ratio of 10∶1. Combined with the results above, this suggested that resistance to CTL cytotoxicity is mediated through other pathways, such as the perforin/granzyme pathway.

### Analysis of Resistance to Granzyme B

Recently, surface expressed cathepsin B has been shown to prevent the action of perforin and thereby preventing the release of granzymes into the target cell cytosol [28]. FACS analysis of the hepatocyte cell lines did not reveal surface expression of cathepsin B, even after IFN-α stimulation (data not shown).

**Figure 7:**
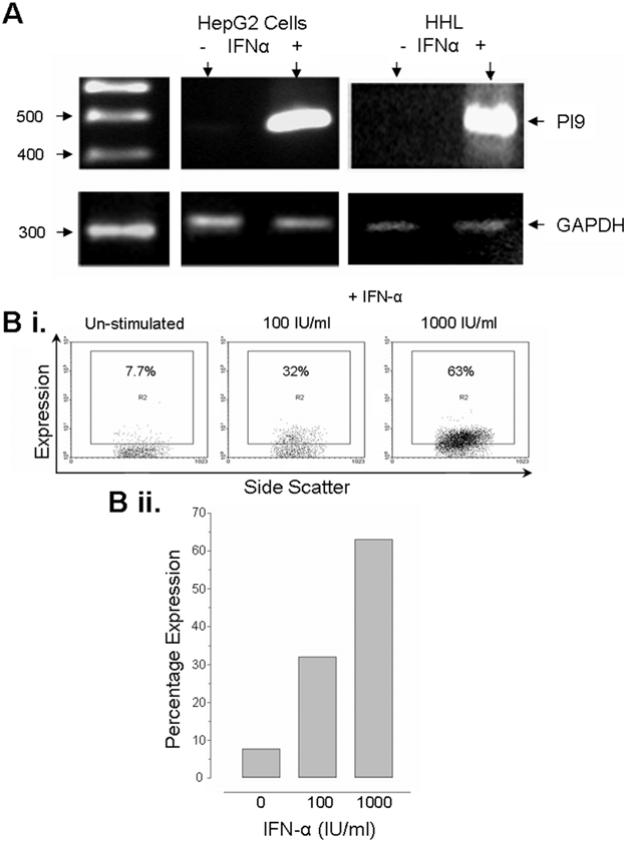
Expression of the granzyme B inhibitor, serine proteinase inhibitor 9 (PI-9). A) RT-PCR revealed that IFN-α stimulated cells, HepG2 and HHL-9 cells, were able to upregulate PI-9. GAPDH expression was analysed as a positive control. Bi and ii) This was confirmed by FACS analysis as described in the methods and materials. Upregulation of PI-9 was shown to be dose dependent.

The granzyme proteinase family consists of many proteinases of which granzyme A and B are thought to play the major roles in inducing target cell apoptosis. Currently, only two naturally expressed inhibitors to these granzymes have been identified. Serine proteinase inhibitor (PI) -9 is known to be a specific inhibitor of granzyme B, while the action of granzyme A has been shown to be inhibited by the pancreatic secretary trypsin inhibitor (PSTI) [29], [30]. Both these inhibitors have been shown to be expressed in rat and murine hepatocytes, as well as human hepatocellular carcinoma lines [31], [32]. RT-PCR confirmed that the HHLs, as well as the hepatocellular carcinoma line, HepG2, were able to up-regulate PI-9 after IFN-α stimulation (Fig. 7A–B). Thus, providing the first evidence that PI-9 expression is not limited in humans to carcinoma derived cell lines.

**Figure 8:**
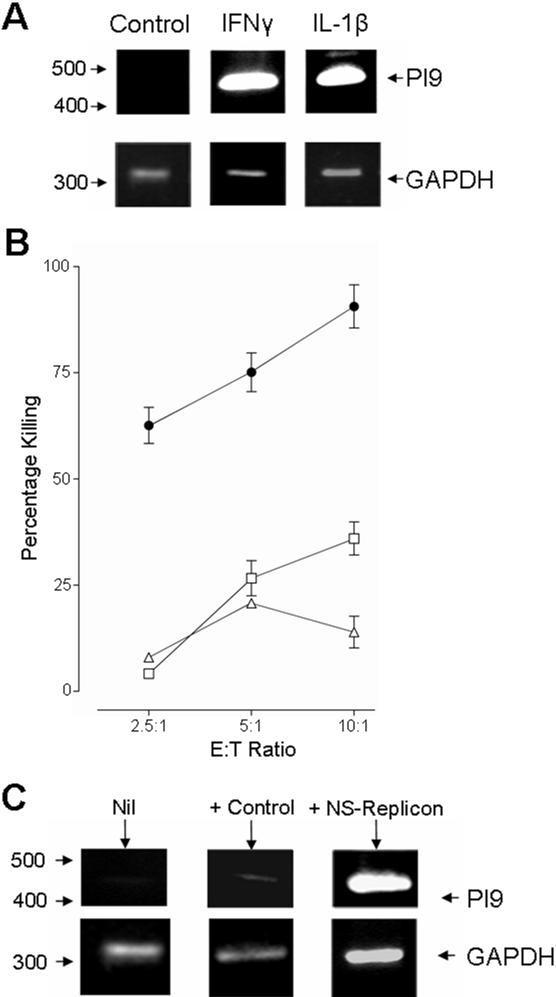
Analysis of alternative triggers for PI-9 expression. A) The up-regulation of PI-9 was also found to be triggered by other inflammatory cytokines. HepG2 cells were treated for 16 hours with either IFN-γ at 100 IU/ml, or IL-1β at 50 ng/ml. GAPDH expression was used as a positive control. B) Stimulation of the HepG2 cell line with either IFN-γ (open squares) or IL-1β (open triangles) also inhibited CTL killing compared to the un-stimulated HepG2 cells (closed circle). C) HepG2 cells were left un-infected (Nil), or infected with either a baculovirus expressing a sub-genomic replicon (NS-replicon), or with a control baculovirus expressing LacZ (+Control). PI-9 expression was analysed by RT-PCR. PI-9 was strongly up-regulated only in the cells expressing the sub-genomic replicon. GAPDH expression was used as a positive control.

To address whether the induction of resistance to killing and the up-regulation of PI-9 were IFN-α specific, we repeated these experiments using alternative inflammatory stimuli: IFN-γ and IL-1β. Both IFN-γ and IL-1β stimulation of HepG2 cell line led to an up-regulation of PI-9 (Fig. 8A), which were accompanied by resistance to CTL killing (Fig. 8B). Furthermore, PI-9 expression could be induced in the HepG2 cell line through infection with a baculovirus that delivered a replicating sub-genomic replicon [33], [34] (Fig. 8C), suggesting the induction of PI-9 expression is not limited to exogenous cytokine stimulation.

### PI.-9 expression *in vivo*

**Figure 9:**
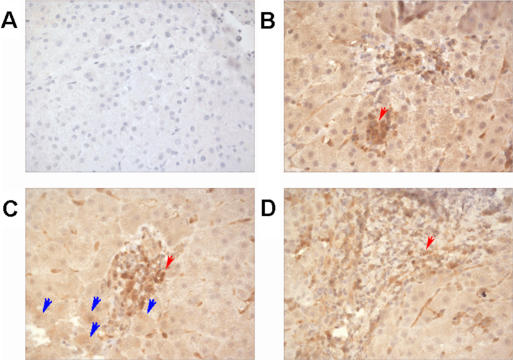
Expression of PI-9 in liver tissue from patients with chronic hepatitis C. Liver specimens obtained from diagnostic biopsy were stained for PI-9 expression as described in the methods. A) Representative negative isotype control. B–D) PI-9 was detected in the majority of hepatocytes. Stronger PI-9 expression, as expected, was seen within the mononuclear infiltrate (highlighted by red arrows), while all the hepatocytes stained positively, albeit at a lower level (the strongest are highlighted by blue arrows).

Finally, to address whether PI-9 expression was an *in vitro* artefact, liver specimens from diagnostic liver biopsies were stained. PI-9 expression was observed in all sections from patients with HCV cirrhosis (n = 3) (Fig. 9). Similar data was obtained from frozen unfixed liver specimens, (data not shown). These data showed for the first time PI-9 expression in primary human hepatocytes *in vivo* during chronic HCV infection.

## Discussion

The ability of hepatocytes to act as good targets for CTLs is crucial for the clearance of heptotrophic viral infections. Central to the success of the CTL responses is the recognition of infected cells through TCR-MHC Class I interactions. The low or absent levels of MHC Class I on hepatocytes have be described *in vivo* and could potentially contribute to the failure of the CTL response to clear HCV [9]–[13]. This study set out to explore this interaction and the influence of up-regulation of MHC Class I by IFN-α.

As expected IFN-α stimulation up-regulated MHC Class I on the hepatocyte derived cell lines. However, unexpectedly this was not accompanied by an increase in sensitivity to CTL cytotoxicity. The IFN-α induced resistance to CTL cytotoxicity was not mediated through alteration of the antigen presentation pathway, as stimulated hepatocyte cell lines were still able to induce IFN-γ expression in CTL. Neither was the resistance due to an inhibition of the FASL-apoptotic pathway. However, IFN-α induced resistance to CTL cytotoxicity was accompanied by the expression of the granzyme B inhibitor–proteinase inhibitor 9 (PI-9).

Hepatocytes have been described by several groups to be highly sensitive to FASL-induced apoptosis, and thus FASL has been presented as the main pathway by which lymphocytes kill hepatocytes [24]–[26]. Jin *et al*. described the ability of liver infiltrating lymphocytes to kill hepatocytes to be predominantly FASL-mediated [35]. If the granzyme pathway is inhibited by cytokine-induction of PI-9 expression within hepatocytes, then the observed sensitivity to FASL could potentially be due to a desensitisation to granzyme B induction of apoptosis.

Granzyme mediated apoptosis primarily involves granzymes A and B. Both of these granzymes have natural inhibitors, the pancreatic secretary trypsin inhibitor (PSTI) and serine protease inhibitor-9 (PI-9) [29], [30]. Both inhibitors have been shown to be expressed in hepatocytes [31], [32]. Here the expression of PI-9 in the hepatocellular carcinoma cell line, HepG2, as well as in immortalised primary human hepatocyte lines (HHL), was confirmed. Moreover, PI-9 was observed for the first time *in vivo*, in liver biopsies from chronically infected individuals. However, the precise contribution PI-9 makes to hepatocyte resistance to CTL cytotoxicity is yet to be clarified. As IFN-α up-regulates a large number of genes, it is probable that other proteins could also be involved in hepatocyte resistance to CTL killing. Knockdown experiments will dissect the specific contribution PI-9 or other inhibitors play.

Regardless of the precise mechanisms behind the hepatocyte resistance to CTL killing, the ability of the hepatocytes to resist killing and promote IFN-γ release could, if this occurs *in vivo*, lead to the skewing of a CTL response towards cytokine-mediated clearance of a hepatic viral infection and away from cytotoxic mechanisms. Such a response has been clearly shown in HBV and LCMV, as well as Duck HBV and Malaria infections where IFN-γ has been shown to be responsible for the clearance of these viruses as opposed to cytopathic mechanisms [14], [15], [36]–[38]. The evolutionary advantage of such a cytoprotective mechanism is not clear, but presumably relates to protection against immunopathology. Very high-level CD8+ T cell responses against a virus infecting large numbers of hepatocytes could readily lead to severe liver damage [39]. This risk of antigen mediated or bystander cellular destruction may be minimised through this pathway.

The ability to resist CD8+ T cell cytotoxicity may be a double-edged sword as such a mechanism could be utilised by infecting viruses to evade CD8+ T cell responses. As long as the virus is able to resist the innate cellular antiviral mechanisms such as PKR, MxA and RNase L, then clearance could only be mediated via the slower FASL pathway and thus contributing to the persistence of the infection.

Further understanding of the mechanisms behind the cytokine-induce hepatocyte resistant to CTL cytotoxicity could lead to novel drug targets, as well as being of potential diagnostic use. However, the full details of the T-cell hepatocyte interaction, while critical to the outcome of many infections, are only just beginning to be addressed.

## Materials and Methods

### Cell lines

HepG2 cells (ECACC) and Human Hepatocyte Lines (HHL), generated by Clayton *et al* [16] -6, -16, -17, were cultured in MEME media (Sigma) plus supplements. Cultures were split once they reached 80% confluence. An EBV transformed B Cell Line (BCL) was cultured in RPMI media (Sigma) with supplements.

Isolation of Peripheral Blood Mononuclear Cells (PBMC): PBMCs were isolated from whole blood collected and mixed with either heparin or EDTA, and separated by density gradient centrifugation over lymphoprep (Nycomed, UK).

### CTL lines

Lines were generated from the PBMC of healthy donors. Approximately 10×10^6^ cells were pulsed with 10 mM specific peptide solution for 1 hour before culturing for 12 –14 days in medium containing IL-2 (50 U/ml). Freshly generated cytotoxic T-cell lines were used in all the assays and were matched to the hepatocyte lines tissue types. CTL lines were generated to match target cells based on their HLA expression. The HHLs expressed HLA-B35 and therefore lines were generated against the HLA-B35 restricted CMV peptide (IPSINVHHY)–labelled as CTL Line 1. As well as expressing HLA-B35 the HepG2 cell line also expressed HLA-A2. CTL lines restricted to the HLA-A2 restricted EBV peptide (GLCTLVAML) were generated and labelled as CTL Line 2.

### Cytokine Stimulation

Cells were seeded in 6 well plates and allowed to settle for 24 hours prior to stimulation for 16 hours under normal culturing conditions. IFN-α (Roche, UK) was used at 1000 IU/ml unless otherwise stated, IFN-γ (R&D, UK) was used at 100 IU/ml, and IL-1β (R&D, UK) was used at 50 ng/ml.

### Cytotoxicity assay (VITAL assay) and rFASL-induced apoptosis

CTL cytotoxicity assays were based on the VITAL assay described by Hermans *et al*. [21] and the FATAL assay described by Sheehy *et al.* [40]. Cognate peptides were pulsed on to the target cells at 10 µg/ml.

### FASL-induced apoptosis

Stimulated or un-stimulated cells were seeded into a 96 U-bottom plate, to which 2 µg/ml of recombinant FAS ligand (FASL)-FLAG (kindly donated by Professor G Screaton, Oxford) was added. This was cross-linked using an anti-FLAG antibody (Abcam, UK), and incubated for 4 hours (unless otherwise indicated) at 37°C. Apoptosis was measured by Annenix V-propidium iodide (BD Pharmigen) staining, or by staining for intracellular activated caspase 3 (as described below).

### Antibodies and Staining, Intercellular Cytokine Staining (ICS)

MHC Class I and CD8 Staining: Cells were harvested and were washed with PBS prior to staining with the anti-MHC Class I (BD Pharmingen, Oxford, UK) or CD8 (BD Pharmingen, Oxford, UK) antibody for 20 minutes at 4°C. For PI-9 (clone 7D8, Stratech Scientific) and IFN-γ (BD Pharmigen, Oxford, UK), cell surface stains were first conducted prior to permeabilisation with BD-PERM (BD Pharmingen, Oxford, UK). The staining for intracellular proteins was then performed using appropriate antibodies as described above.

### Antigen Presentation Assay

CTL effector (E) cells were stimulated by addition of the specific peptide, target (T) cells pulsed with the specific peptide (E∶T 10∶1), or by PMA (10 ngm/ml) ionomycin (1 µg/ml) (Sigma). The cells were incubated for 1 hour at 37°C before the addition of Golgi Stop™ (BD Pharmingen). The cells were then left for a further 4 hours at 37°C before staining for CD8 and IFN-γ (as described above).

### Immunohistology

The expression of PI-9 in liver tissue was detected by immunostaining using monoclonal PI- 9 antibody (Stratech Scientific, Soham, UK) at 10 µg/ml as a primary antibody and Dako EnVision detection kit (DAKO, Ely, UK). All sections were stained in parallel with an unrelated primary antibody (MR12) to provide a negative isotype control. Formalin-fixed, paraffin-embedded liver biopsy specimens from 3 HCV patients were obtained from the archive of the Department of Pathology at the John Radcliffe Hospital, Oxford, UK. Table 1 shows the patient clinical data. Informed written patient consent was obtained for each biopsy, which were taken for clinical staging of the disease. Ethical approval for the study was obtained from the local ethical review panel (COREC) and the protocols conformed to the ethical guidelines of the 1975 Declaration of Helsinki.

**Table 1:** Clinical data of HCV patients at time of biopsy.

Patient ID	Sex	Age (years)	Genotype	Fibrosis (Ishak score )/6	Periportal or Periseptal Interface Hepatitis/4	Confluent Necrosis/6	Lobular Inflammation/4	Portal Inflammation/4	Bilirubing/L	Alkaline Phosphatase IU/L	Albumin g/L	Prothrombin g/L	Alanine Aminotransferase (ALT)IU/L
1	F	43	2a/2c	6	1	0	1	2	10	159	44	13.2	115
2	M	39	3a	5	1	1	2	3	11	203	43	12.2	94
3	M	43	1a	6	1	0	0	2	24	189	41		139

### RT-PCR

RNA was extracted from the cells using the RNAeasy kit (Qiagen). A cDNA library was generated using oligo-T primers. PI-9 mRNA was amplified using Forward- CTGCCCTGGCCATGGTTCTCCTA and Reverse-CTGGCCTTTGCTCCTCCTGGTTTA primers, for 32 cycles with an annealing temperature of 64°C. As a control, GAPDH was amplified from the cDNA library using Forward- GGTCGGAGTCAACGGATTTG and Reverse- ATGAGC CCCAGCCTTCTCCAT primers, for 16 cycles and an annealing temperature of 61°C.

## References

[1] (1999). Hepatitis C–global prevalence (update).. Wkly Epidemiol Rec.

[2] Lechner F, Cuero AL, Kantzanou M, Klenerman P (2001). Studies of human antiviral CD8+ lymphocytes using class I peptide tetramers.. Rev Med Virol.

[3] Lechner F, Gruener NH, Urbani S, Uggeri J, Santantonio T (2000). CD8+ T lymphocyte responses are induced during acute hepatitis C virus infection but are not sustained.. Eur J Immunol.

[4] Lechner F, Wong DK, Dunbar PR, Chapman R, Chung RT (2000). Analysis of successful immune responses in persons infected with hepatitis C virus.. J Exp Med.

[5] Calne RY (1969). “Strange english PIGS”.. Lancet.

[6] Calne RY, Sells RA, Pena JR, Davis DR, Millard PR (1969). Induction of immunological tolerance by porcine liver allografts.. Nature.

[7] Limmer A, Ohl J, Kurts C, Ljunggren HG, Reiss Y (2000). Efficient presentation of exogenous antigen by liver endothelial cells to CD8+ T cells results in antigen-specific T-cell tolerance.. Nat Med.

[8] Bertolino P, McCaughan GW, Bowen DG (2002). Role of primary intrahepatic T-cell activation in the ‘liver tolerance effect’.. Immunol Cell Biol.

[9] Daar AS, Fuggle SV, Fabre JW, Ting A, Morris PJ (1984). The detailed distribution of HLA-A, B, C antigens in normal human organs.. Transplantation.

[10] Eisenberger CF, Viebahn R, Lauchart W, de Groot H, Becker HD (1994). MHC antigen presentation on the surface of hepatocytes: modulation during and after hypoxic stress.. Transpl Int.

[11] Gugenheim J, Reynes M, Crafa F, Saint-Paul MC, Fabiani B (1996). Normothermic ischemia induces major histocompatibility complex class I expression in hepatocytes.. Eur Surg Res.

[12] So SK, Platt JL, Ascher NL, Snover DC (1987). Increased expression of class I major histocompatibility complex antigens on hepatocytes in rejecting human liver allografts.. Transplantation.

[13] Lau JY, Bird GL, Naoumov NV, Williams R (1993). Hepatic HLA antigen display in chronic hepatitis B virus infection. Relation to hepatic expression of HBV genome/gene products and liver histology.. Dig Dis Sci.

[14] Guidotti LG, Borrow P, Brown A, McClary H, Koch R (1999). Noncytopathic clearance of lymphocytic choriomeningitis virus from the hepatocyte.. J Exp Med.

[15] Guidotti LG, Rochford R, Chung J, Shapiro M, Purcell R (1999). Viral clearance without destruction of infected cells during acute HBV infection.. Science.

[16] Clayton RF, Rinaldi A, Kandyba EE, Edward M, Willberg C (2005). Liver cell lines for the study of hepatocyte functions and immunological response.. Liver Int.

[17] Bertoletti A, Costanzo A, Chisari FV, Levrero M, Artini M (1994). Cytotoxic T lymphocyte response to a wild type hepatitis B virus epitope in patients chronically infected by variant viruses carrying substitutions within the epitope.. J Exp Med.

[18] Chisari FV (1997). Cytotoxic T cells and viral hepatitis.. J Clin Invest.

[19] Timm J, Lauer GM, Kavanagh DG, Sheridan I, Kim AY (2004). CD8 epitope escape and reversion in acute HCV infection.. J Exp Med.

[20] Theofilopoulos AN, Baccala R, Beutler B, Kono DH (2005). Type I interferons (alpha/beta) in immunity and autoimmunity.. Annu Rev Immunol.

[21] Hermans IF, Silk JD, Yang J, Palmowski MJ, Gileadi U (2004). The VITAL assay: a versatile fluorometric technique for assessing CTL- and NKT-mediated cytotoxicity against multiple targets in vitro and in vivo.. J Immunol Methods.

[22] Jensen ER, Glass AA, Clark WR, Wing EJ, Miller JF (1998). Fas (CD95)-dependent cell-mediated immunity to Listeria monocytogenes.. Infect Immun.

[23] Kafrouni MI, Brown GR, Thiele DL (2001). Virally infected hepatocytes are resistant to perforin-dependent CTL effector mechanisms.. J Immunol.

[24] Li XK, Fujino M, Sugioka A, Morita M, Okuyama T (2001). Fulminant hepatitis by Fas-ligand expression in MRL-lpr/lpr mice grafted with Fas-positive livers and wild-type mice with Fas-mutant livers.. Transplantation.

[25] Ogasawara J, Watanabe-Fukunaga R, Adachi M, Matsuzawa A, Kasugai T (1993). Lethal effect of the anti-Fas antibody in mice.. Nature.

[26] Song E, Lee SK, Wang J, Ince N, Ouyang N (2003). RNA interference targeting Fas protects mice from fulminant hepatitis.. Nat Med.

[27] Adams JM, Cory S (1998). The Bcl-2 protein family: arbiters of cell survival.. Science.

[28] Balaji KN, Schaschke N, Machleidt W, Catalfamo M, Henkart PA (2002). Surface cathepsin B protects cytotoxic lymphocytes from self-destruction after degranulation.. J Exp Med.

[29] Sun J, Bird CH, Sutton V, McDonald L, Coughlin PB (1996). A cytosolic granzyme B inhibitor related to the viral apoptotic regulator cytokine response modifier A is present in cytotoxic lymphocytes.. J Biol Chem.

[30] Tsuzuki S, Kokado Y, Satomi S, Yamasaki Y, Hirayasu H (2003). Purification and identification of a binding protein for pancreatic secretory trypsin inhibitor: a novel role of the inhibitor as an anti-granzyme A.. Biochem J.

[31] Barrie MB, Stout HW, Abougergi MS, Miller BC, Thiele DL (2004). Antiviral cytokines induce hepatic expression of the granzyme B inhibitors, proteinase inhibitor 9 and serine proteinase inhibitor 6.. J Immunol.

[32] Uda K, Murata A, Nishijima J, Doi S, Tomita N (1994). Elevation of circulating monitor peptide/pancreatic secretory trypsin inhibitor-I (PSTI-61) after turpentine-induced inflammation in rats: hepatocytes produce it as an acute phase reactant.. J Surg Res.

[33] McCormick CJ, Challinor L, Macdonald A, Rowlands DJ, Harris M (2004). Introduction of replication-competent hepatitis C virus transcripts using a tetracycline-regulable baculovirus delivery system.. J Gen Virol.

[34] McCormick CJ, Rowlands DJ, Harris M (2002). Efficient delivery and regulable expression of hepatitis C virus full-length and minigenome constructs in hepatocyte-derived cell lines using baculovirus vectors.. J Gen Virol.

[35] Jin Y, Fuller L, Carreno M, Zucker K, Roth D (1997). The immune reactivity role of HCV-induced liver infiltrating lymphocytes in hepatocellular damage.. J Clin Immunol.

[36] Guidotti LG, McClary H, Loudis JM, Chisari FV (2000). Nitric oxide inhibits hepatitis B virus replication in the livers of transgenic mice.. J Exp Med.

[37] Schofield L, Ferreira A, Altszuler R, Nussenzweig V, Nussenzweig RS (1987). Interferon-gamma inhibits the intrahepatocytic development of malaria parasites in vitro.. J Immunol.

[38] Schultz U, Chisari FV (1999). Recombinant duck interferon gamma inhibits duck hepatitis B virus replication in primary hepatocytes.. J Virol.

[39] Ehl S, Klenerman P, Zinkernagel RM, Bocharov G (1998). The impact of variation in the number of CD8(+) T-cell precursors on the outcome of virus infection.. Cell Immunol.

[40] Sheehy ME, McDermott AB, Furlan SN, Klenerman P, Nixon DF (2001). A novel technique for the fluorometric assessment of T lymphocyte antigen specific lysis.. J Immunol Methods.

